# Physiological Responses to Commanded Terrain Conditions in a Real-World Video-Based Indoor Cycling System

**DOI:** 10.3390/sports14100460

**Published:** 2026-10-16

**Authors:** Sang Keun You, Mi Yu, Tae Kyu Kwon

**Affiliations:** 1 Department of Healthcare Engineering, Graduate School, Jeonbuk National University, Jeonju 54896, Republic of Korea; dbtkdrms9742@gmail.com; 2 Division of Biomedical Engineering, College of Engineering, Jeonbuk National University, Jeonju 54896, Republic of Korea; yumi@jbnu.ac.kr; 3 Research Center of Healthcare & Welfare Instrument for the Aged, Jeonbuk National University, Jeonju 54896, Republic of Korea

**Keywords:** indoor cycling, heart rate variability, respiratory gas analysis, terrain-driven resistance control, exercise intensity, digital fitness, physiological characterization

## Abstract

**Background and Purpose:** Indoor cycling systems can combine real-world riding footage with resistance commands derived from terrain data. This study characterized cardiovascular, heart rate variability, and metabolic responses to three commanded terrain conditions. **Methods:** Analyses included three 30 min sessions per participant in 30 healthy men (22 ± 2 years) under low (predominantly downhill), moderate (mixed climbing and descending), and high (predominantly uphill) terrain conditions. All participants completed the three conditions across three study visits in a fixed order: moderate, low, and high. Participants were instructed to maintain 50–60 rpm. Heart rate (HR) and heart rate variability (HRV) were analyzed in 30 participants, and respiratory gas exchange in 29. Condition and measurement period effects were assessed using two-way repeated-measures analysis of variance; all four HRV indices were natural-log-transformed. In exploratory analyses of the moderate condition, participant-level HR, oxygen uptake (VO_2_), and carbon dioxide output (VCO_2_) slopes were compared between climbing and descending segments defined from an approximately time-aligned reference terrain profile. **Results:** HR, respiratory frequency, VO_2_, VCO_2_, and metabolic equivalents increased across conditions, whereas the time-domain HRV indices SDNN and RMSSD decreased (all condition effects, *p* < 0.01). Exploratory frequency-domain analyses also showed decreases in LF and HF across conditions; interpretation remains limited by exercise nonstationarity, respiration, and unassessed sensitivity to recording quality. In the moderate condition, median HR slopes were +6.34 bpm/min during classified climbing segments and −2.32 bpm/min during classified descending segments (*p* = 0.005; rank-biserial *r_rb_* = 0.58); the climbing slope exceeded the descending slope in 70% of participants. Corresponding differences were observed for VO_2_ (+126 vs. −102 mL·min^−2^; *p* = 0.016) and VCO_2_ (+132 vs. −121 mL·min^−2^; *p* = 0.004). **Conclusions:** Physiological responses differed across the three commanded terrain conditions under the fixed-order protocol, with group-level differences between classified climbing and descending segments in the moderate condition. Delivered mechanical workload was not validated, participant-specific terrain alignment was approximate, and condition effects could not be separated from visit-order effects. These findings characterize responses under the tested protocol and do not establish mechanical load accuracy or precise terrain tracking.

## 1. Introduction

Physical inactivity is an independent risk factor for major chronic diseases, including cardiovascular disease, type 2 diabetes, obesity, and sarcopenia [1,2]. To promote health, the World Health Organization (WHO) recommends that adults perform 150–300 min of moderate-intensity aerobic physical activity or 75–150 min of vigorous-intensity aerobic physical activity per week [3]. Indoor cycling is an aerobic exercise modality that can be performed irrespective of weather or geographic constraints. Because it is non-weight-bearing and imposes relatively little stress on the joints, it has been widely used in clinical rehabilitation and health-promotion programs [4,5]. More recently, video-based riding content, digital exercise interventions, and real-time biosignal monitoring have been integrated into indoor cycling. Consequently, indoor cycling systems are evolving toward platforms that link exercise performance and physiological responses in real time. From this perspective, such a platform can be conceptualized as a human-machine system (HMS) [6].

During outdoor cycling, environmental factors such as road gradient, rolling resistance arising from the road surface, and aerodynamic drag act in combination. These factors determine the required mechanical power output and can be quantified using mathematical models of cycling dynamics [7]. In particular, increasing road gradient changes the intensity and recruitment pattern of lower-limb muscle activity [8,9]. The resulting increases in muscular activity and metabolic demand are accompanied by greater oxygen uptake and altered autonomic regulation, ultimately producing cardiovascular responses [10]. Therefore, an indoor exercise system intended to reproduce outdoor terrain should do more than provide visual immersion. Such systems should be designed to provide terrain-dependent exercise demands that are associated with appropriate and measurable physiological responses.

The quantitative control of exercise intensity is a prerequisite for individualized exercise prescription. The American College of Sports Medicine (ACSM) guidelines emphasize the precise control of exercise intensity, frequency, and duration to improve cardiorespiratory fitness [5]. Standardized terminology for exercise intensity has also recently been proposed to improve comparability across studies [11]. The importance of quantitative intensity control is even greater in telerehabilitation and digital exercise interventions because safety and efficacy cannot be assured if a system fails to reliably deliver the intended intensity [4]. A previous study evaluated a similar interactive indoor cycling device using real-world video in 20 patients with cardiovascular disease [12]. In a single 10 min session, the device was compared with treadmill cardiopulmonary exercise testing (CPET). The device elicited exercise within the intensity range recommended for cardiac rehabilitation, supporting its short-term feasibility and safety. However, that study focused on feasibility and safety during a single exercise session and did not examine whether physiological responses differed by terrain difficulty or whether physiological changes tracked the direction of terrain load, such as climbing versus descending.

Many current video-based indoor cycling and virtual reality (VR) exercise platforms have primarily focused on improving visual immersion, motivation, enjoyment, and usability [13,14]. Research on exergames has also highlighted limitations that arise when the physical load is insufficient. When physical loading is weak relative to visual stimulation, exercise intensity may remain only light to moderate, making it difficult to induce a consistent aerobic training effect [15]. Exercise intensity on these platforms has often been evaluated only using summary measures such as heart rate, energy expenditure, and rating of perceived exertion (RPE) [16,17,18]. Consequently, there has been limited validation of the extent to which system-controlled loading corresponds to users’ physiological responses during exercise. Visual information itself can also modulate physiological and perceptual responses. Previous research has shown that optic-flow speed affects perceived exertion and self-selected power output [19] and may alter heart rate, even when dissociated from subjective effort [20]. These findings indicate that visual content alone does not guarantee the intended physiological load. It is therefore necessary to determine, using objective biosignals, whether different commanded terrain conditions are accompanied by distinguishable physiological responses.

The design rationale is also supported by cognitive neuroscientific evidence regarding congruence between visual information and bodily sensation. Sensory integration is facilitated when multisensory information is consistent [21]. Through internal forward models, the central nervous system anticipates the exercise load implied by visual information [22,23]. If the actual resistance differs from this prediction, the visuo-haptic mismatch may be processed as a sensory prediction error [24]. VR cycling studies have experimentally manipulated congruence and incongruence between visual slope and pedal resistance, suggesting that perceived effort may contribute to cardiovascular regulation [25,26].

Commercial virtual cycling platforms provide relevant comparators. Zwift supports gradient-dependent resistance with compatible controllable trainers, with the Trainer Difficulty setting adjusting the strength of this relationship [27]. Published studies have used Zwift for performance testing and physiological assessment. Matta et al. reported reproducible mean power output across three home-based 20 min time trials in 44 cyclists [28]. Contreras-Briceño et al. validated a respiratory wearable device in 17 adults and subsequently used Zwift-supported ramp tests in 107 recreational cyclists to estimate ventilatory thresholds outside the laboratory [29]. These studies provide precedents for remote testing and biosignal assessment; they did not validate the terrain control accuracy of the present system. Our implementation combines recorded first-person riding videos with a GPX- and structure-from-motion-based terrain pipeline and resistance commands delivered through an integrated actuator. The present study characterizes HR, HRV, and respiratory gas responses across three commanded conditions and within reference-defined climbing and descending segments. Terrain-linked resistance is an established capability; the contribution here concerns its implementation and physiological characterization.

The purpose of this study was to characterize physiological responses to the commanded terrain conditions of a dynamic resistance indoor cycling system. Cardiovascular responses, heart rate variability, and metabolic variables were evaluated using multiple biosignals. First, condition-level comparisons examined whether physiological responses differed across the selected low, moderate, and high commanded terrain conditions. Second, segment-level analyses examined whether participant-level HR, VO_2_, and VCO_2_ slopes differed between intervals classified as climbing and descending in the moderate condition. The use of slopes and participant-level summaries was intended to reduce reliance on absolute signal levels, which are subject to time-dependent influences during prolonged exercise, including possible cardiovascular drift [30], and to shared trends in nonstationary time series [31,32]. Because the delivered mechanical power was not validated and individual route positions were not recorded continuously, the segment analysis relied on an approximately aligned reference terrain profile. The study, therefore, provides a physiological characterization of the tested conditions rather than validating the delivered mechanical workload or the specific benefit of visual–mechanical synchronization.

## 2. Materials and Methods

### 2.1. Real-World Video-Based Indoor Cycling System

The indoor cycling platform was custom-built by Tanda, Jeonju, Republic of Korea. It was designed to automatically adjust resistance based on terrain data, with visual progression and resistance commands linked to the same terrain events. The physiological evaluation characterized responses to commanded terrain conditions; it did not independently validate delivered mechanical workload or isolate the specific effect of visual–mechanical congruence.

#### 2.1.1. Hardware Interface and Dynamic Resistance Actuator

**Figure 1:**
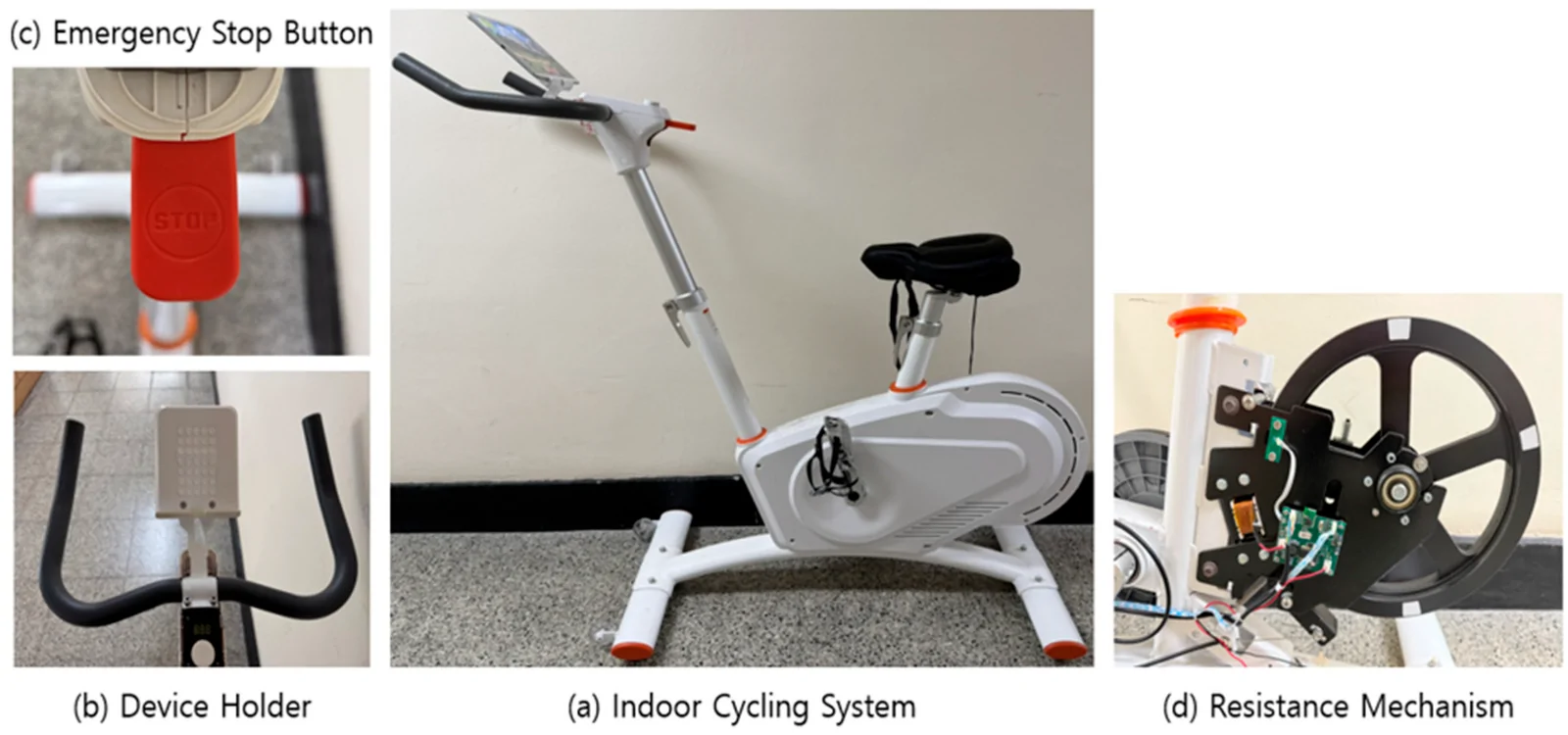
Structure and major components of the real-world video-based indoor cycling system. The system includes an indoor cycle, a mobile device holder, an emergency stop mechanism, and an electromagnetic resistance actuator.

The mechanical structure of the system was designed to accommodate users with a range of anthropometric characteristics. The principal specifications are presented in Table 1 and Figure 1. The system measured 975 (L) × 520 (W) × 1030 (H) mm and weighed 30 kg. The specified maximum torque, power output, and cadence were 32 N·m, 402 W, and 120 rpm, respectively. It accommodated users 155–185 cm tall and up to 115 kg in body mass, and reported cadence and power. However, the power values obtained during this experiment were calculated by software and were not validated as measures of delivered mechanical workload; therefore, they were excluded as an analytical load metric (Section 4.6). A mobile-device-holder and an integrated display were mounted on the handlebar assembly. The display enabled the monitoring of system communication and hardware control status. To reduce the risk of injury during exercise, a mechanical emergency stop mechanism was installed beneath the handlebar assembly (Figure 1c). In an emergency, the mechanism stopped the 7 kg steel flywheel by applying a friction brake pad. The drive unit incorporated an electromagnetic dynamic resistance actuator (Figure 1d). During riding, terrain gradient data were transmitted from the application to the actuator via Bluetooth Low Energy to adjust the electromagnetic field and eddy current resistance applied to the flywheel. Target resistance values corresponding to the terrain gradient were sent to the actuator via feedforward control [33]. The application used cadence and software-reported power to determine movement speed in the virtual environment. The system thus formed a closed-loop architecture linking terrain input, resistance commands, user exercise response, and virtual movement speed.

#### 2.1.2. Terrain Data Pipeline and Visual–Mechanical Synchronization

**Figure 2:**
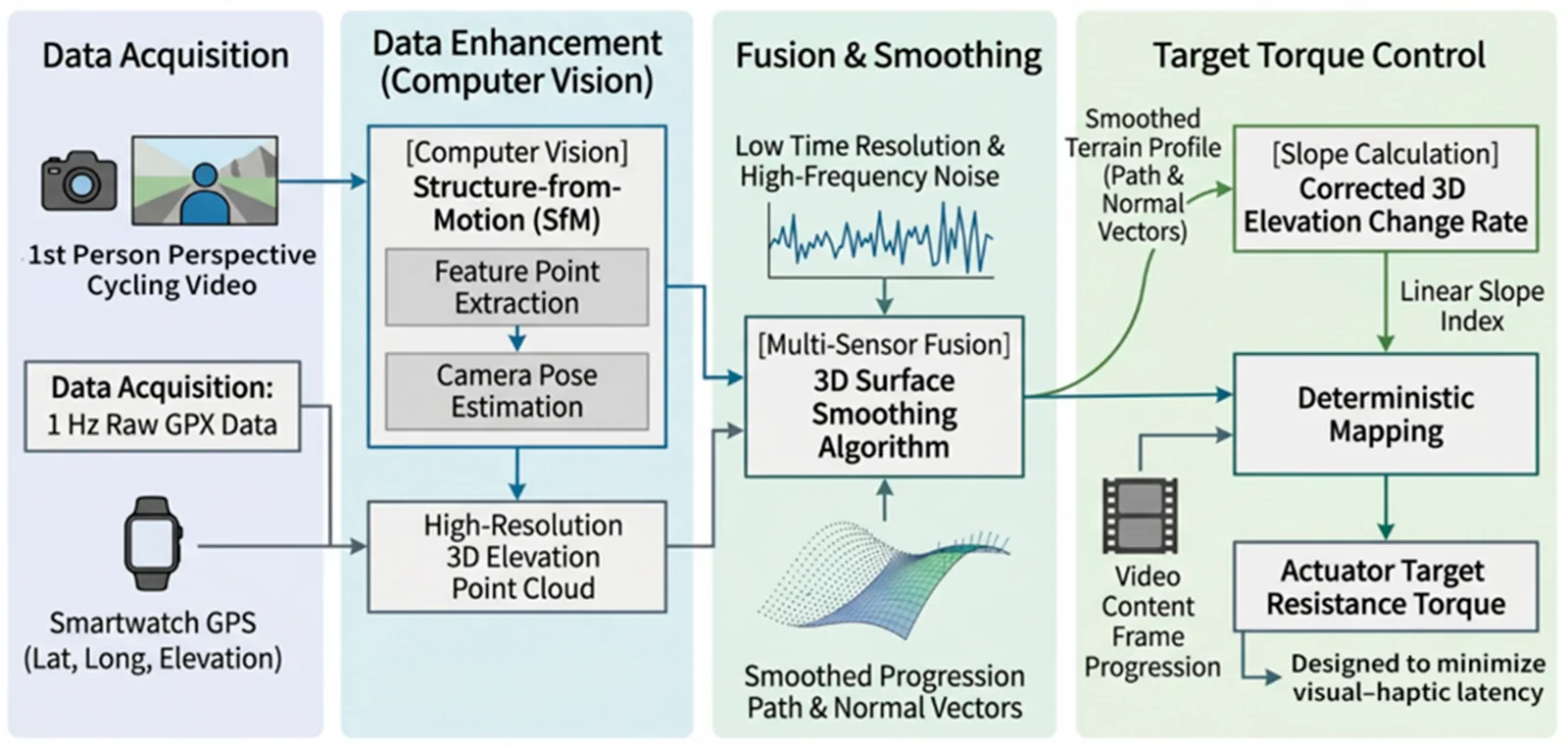
Terrain data pipeline and visual–mechanical synchronization process.

To link visual content to resistance commands, the system incorporated a multisensor fusion pipeline for terrain data (Figure 2). During data acquisition, a first-person riding video was recorded while raw GPS Exchange Format (GPX) data were simultaneously collected at 1 Hz using the GPS module of a smartwatch (Fenix 6S; Garmin, Kansas, MI, USA). The GPX data included latitude, longitude, and altitude. To obtain a more detailed terrain representation, structure-from-motion (SfM) [34] was applied to extract frame-level features and estimate camera pose, producing a high-resolution three-dimensional elevation profile. A three-dimensional surface-smoothing algorithm was then applied to reduce high-frequency noise in the raw GPS signal and limit abrupt changes in the resistance command [35]. The corrected three-dimensional elevation change rate was converted into a linear slope index that determined the actuator’s target resistance torque as the video progressed. This pipeline was intended to coordinate visual progression with resistance commands; the present analysis focused on physiological responses to the commanded terrain conditions rather than quantifying visual–mechanical latency or delivered torque.

#### 2.1.3. Cyber–Physical Indoor Cycling Application

**Figure 3:**
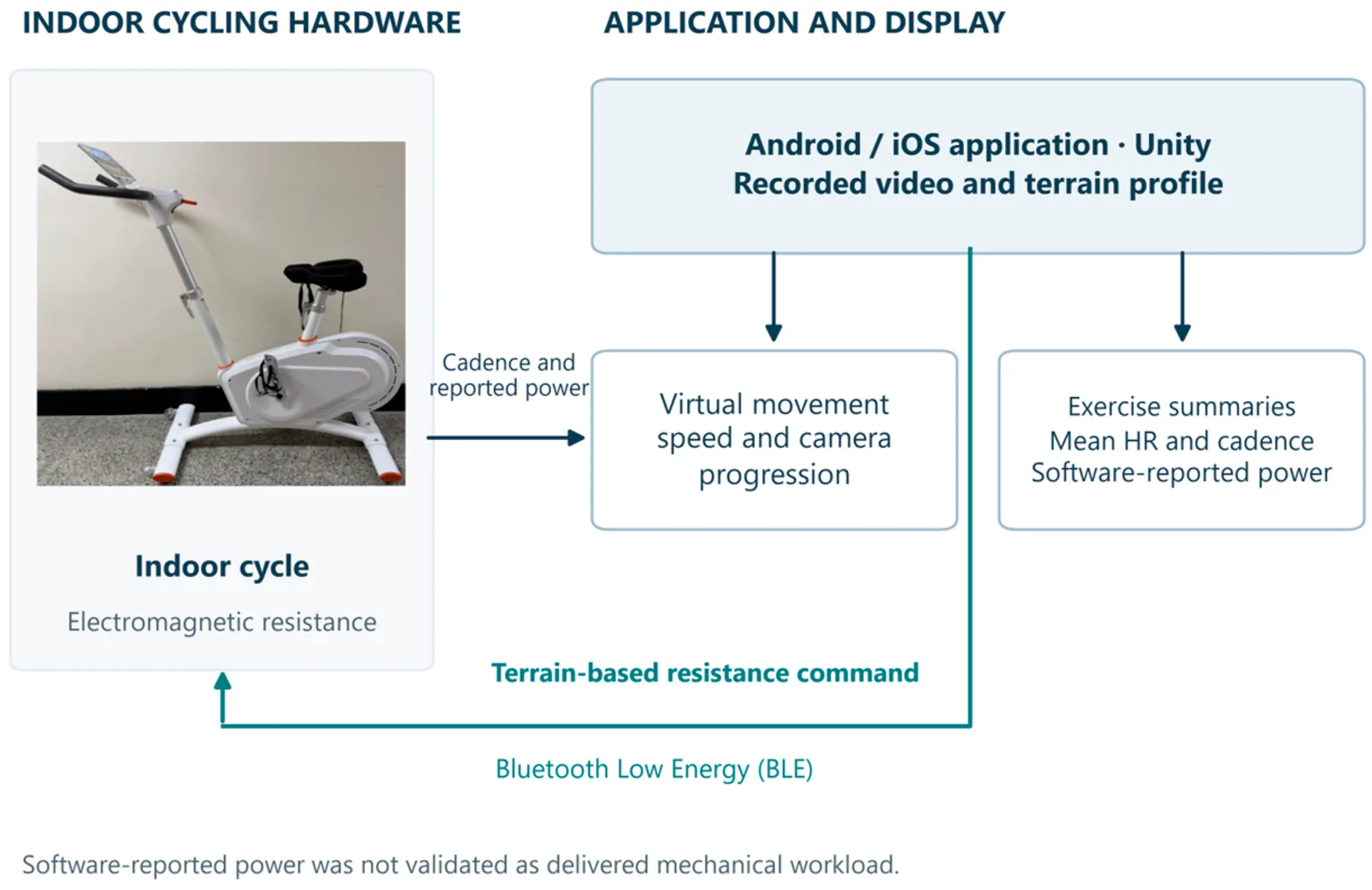
Indoor cycling hardware and application architecture. The photograph shows the experimental indoor cycle. The application uses cadence and software-reported power to update virtual movement speed and camera progression, and transmits terrain-based resistance commands to the electromagnetic actuator via Bluetooth Low Energy (BLE). Software-reported power was not independently validated as the delivered mechanical workload.

The system software was implemented as Android- and iOS-based applications that connected the hardware to the virtual environment (Figure 3). Once bidirectional communication with the hardware was established, cadence (rpm) and software-reported power data were acquired in real time. These data were processed using rigid-body dynamics calculations within the Unity rendering engine (version 2020.3.22f1) to update the virtual avatar’s three-dimensional movement speed and the first-person camera’s position. The software also generated exercise summaries, including mean heart rate, cadence, and reported power. Detailed participant-level records of virtual movement speed were unavailable for analysis. Consequently, condition-specific virtual cycling speeds (km/h) could not be reported. The physiological evaluation employed terrain-based resistance commands and biosignal collection functions. Software-reported power was not independently validated and was therefore excluded as a measure of delivered mechanical workload (Section 4.6).

#### 2.1.4. Experimental Stimuli and Difficulty Classification

**Figure 4:**
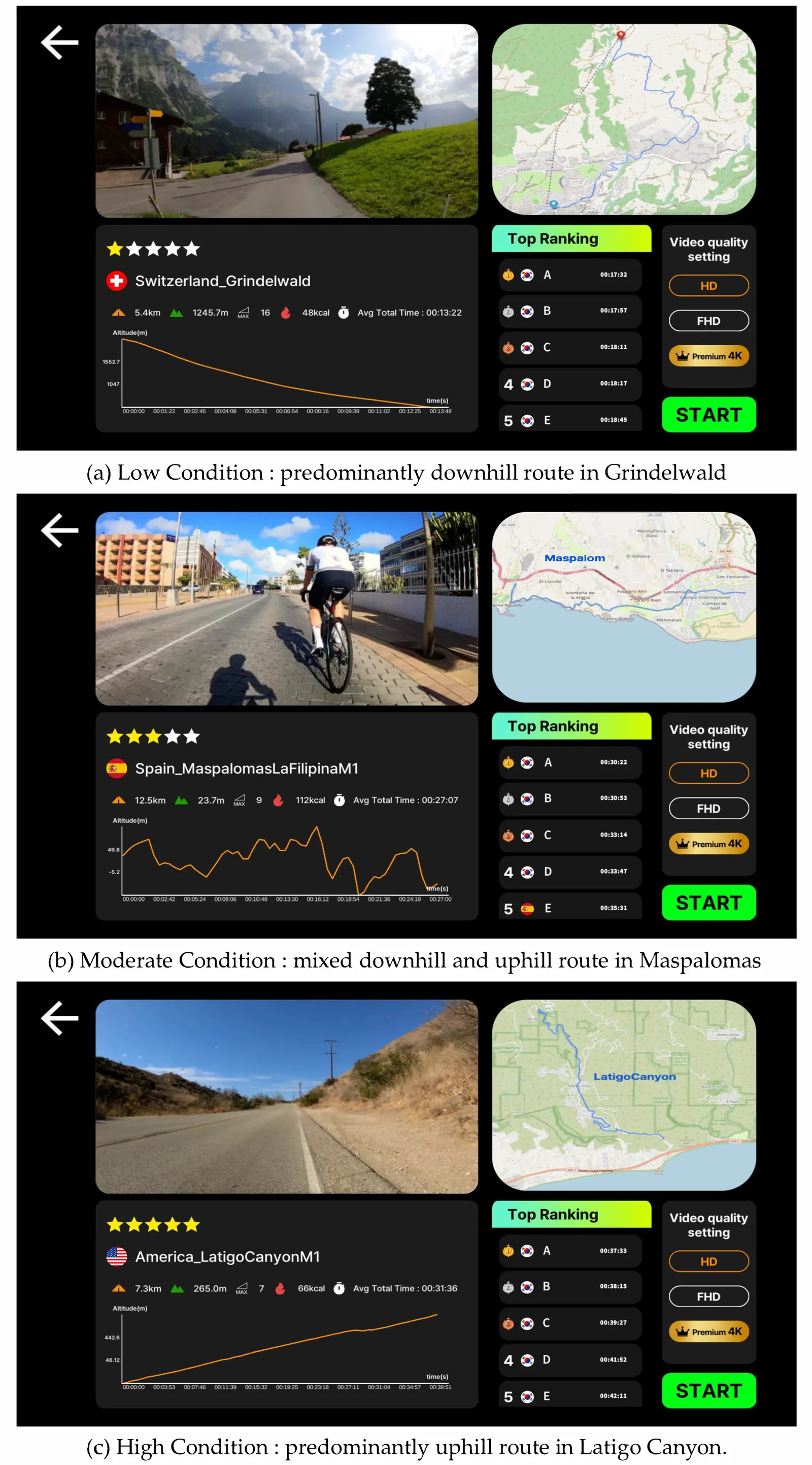
Experimental content used for the low, moderate, and high conditions. The arrow indicates the back-navigation button, and the stars represent the content intensity displayed in the application interface.

To evaluate physiological responses across system-defined terrain conditions, a library of real-terrain video content was constructed as the experimental stimulus set. The library comprised 62 videos recorded on actual terrain in Korea and other countries, including 8 domestic and 54 international routes. Content classification considered cumulative elevation gain, mean gradient, total riding distance, and estimated completion time. Five difficulty levels were defined: very easy (*n* = 5), easy (*n* = 19), moderate (*n* = 13), difficult (*n* = 10), and very difficult (*n* = 15). Three videos were selected for the physiological evaluation to represent low-, moderate-, and high-commanded terrain-load conditions. These condition labels were based on terrain composition: predominantly downhill for the low condition; mixed downhill, level, and uphill sections, with a relatively high proportion of climbing, for the moderate condition; and predominantly uphill for the high condition (Figure 4). The labels describe the selected terrain conditions rather than independently verified mechanical work rates or participant-specific relative exercise intensities.

### 2.2. Physiological Evaluation

#### 2.2.1. Participants

Thirty healthy young adult men were recruited to reduce age- and sex-related heterogeneity in this initial physiological characterization. Age was calculated from self-reported dates of birth. Mean age was 22 ± 2 years (range, 18–27 years), mean height was 176 ± 6 cm, and mean body mass was 77 ± 11 kg. No participant had a history of cardiovascular or respiratory disease or a condition that limited exercise performance. In a questionnaire completed after the measurement sessions, 25 of 30 participants (83.3%) reported exercising regularly. Five participants (16.7%) listed cycling as a regular or home-based exercise activity. These self-reports did not provide objective measures of aerobic fitness or a standardized classification of cycling training status. The recruitment target was 30 participants. Previous studies on spinning and cycling were consulted when setting this target [36,37]. Seo [36] investigated a 12-week spinning intervention in 16 women with anemia, while Chin and Lee [37] reported a study sample of 34 obese male college students undergoing an 8-week cycling intervention. These studies provided examples of participant numbers in related exercise research. A separate sensitivity analysis was performed using G*Power version 3.1.9.7 with the ANOVA: Repeated measures, within the factors option. With one group, three repeated measurements, a total sample size of 30, a nominal significance level of α = 0.05, a target power of 0.80, an assumed correlation of 0.50 among repeated measurements, and a nonsphericity correction of ε = 1, the minimum detectable effect size was approximately Cohen’s f = 0.237. This result characterizes sensitivity for the specified one-factor repeated-measures model under these assumptions. The sample size was not determined specifically for the within-participant climbing–descending contrast; these segment-level analyses are interpreted as exploratory. The study was approved by the Jeonbuk National University Institutional Review Board (IRB No. JBNU 2024-09-007-001). All procedures complied with the ethical principles of the Declaration of Helsinki. The study objectives, procedures, and use of video recording were explained in advance. Written informed consent was obtained after participants confirmed their voluntary willingness to participate.

#### 2.2.2. Experimental Design and Protocol

A repeated-measures design was used. Each participant attended three study visits in total, completing one condition per visit in the fixed order: moderate, low, and high at visits 1, 2, and 3, respectively. Inter-visit intervals were calculated from the actual session start times recorded in the measurement data. Each of the 30 participants contributed two intervals, between visits 1 and 2 and between visits 2 and 3, yielding 60 intervals in total. The inter-visit interval was 53.9 ± 49.9 h (mean ± SD; range, 17.4–285.6 h). Each exercise session lasted 30 min. Participants were instructed to maintain a pedaling cadence of 50–60 rpm in all conditions. No separate pre-experiment familiarization session was conducted. Participants were instructed to avoid caffeine, alcohol, and strenuous exercise from the day before each experimental session. No specific meal restrictions were imposed. Recorded start times for the 90 cycling sessions ranged from 09:59 to 17:07. The within-participant range in start time across the three conditions was 3.0 ± 1.9 h (mean ± SD).

**Figure 5:**
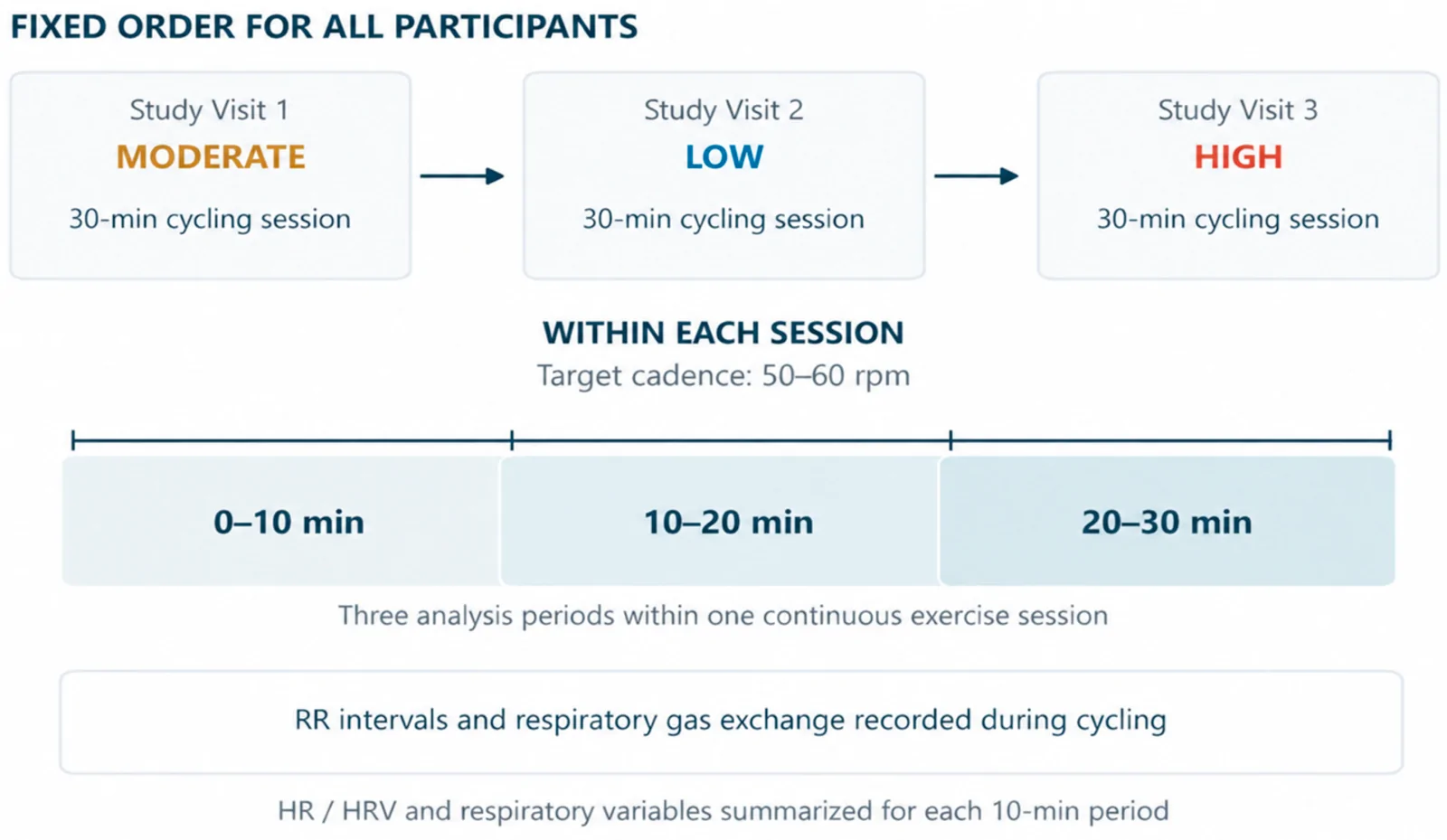
Experimental protocol for the three study visits. All participants completed the moderate, low, and high conditions at visits 1, 2, and 3, respectively, in a fixed order. Each visit included a single continuous 30 min cycling session, divided into three 10 min analysis periods. Participants were instructed to maintain a target cadence of 50–60 rpm.

The cadence target was intended to limit the contribution of voluntary pacing changes to differences in metabolic demand. Real-time rpm was displayed at the top of the system screen, and participants were instructed to monitor the display continuously and maintain the prescribed range. Session-mean cadence values used in this study were obtained from the system’s results screen displayed at the end of each exercise session. Continuous cadence recordings for individual participants were not available for analysis. The proportion of exercise time within the prescribed range and cadence during the terrain-defined segments, therefore, could not be quantified. The overall experimental design is shown in Figure 5.

Content difficulty was determined from terrain composition. Predominantly downhill content from Grindelwald was used for the low condition (Figure 4a). Mixed downhill and uphill content from Maspalomas was used for the moderate condition (Figure 4b). Predominantly uphill content from Latigo Canyon was used for the high condition (Figure 4c).

#### 2.2.3. Physiological Measurements and Equipment

Physiological signals were recorded during the 30 min exercise sessions. Beat-to-beat RR intervals for heart rate and heart rate variability analyses were recorded using a chest-worn heart rate sensor (Polar H10; Polar Electro Inc., Bethpage, NY, USA). Respiratory frequency, oxygen uptake (VO_2_), carbon dioxide output (VCO_2_), and metabolic equivalents (METs) were measured using a respiratory gas analysis system (Quark CPET; COSMED S.r.l., Albano Laziale, Italy). Respiratory gas exchange was measured using the Quark CPET in breath-by-breath mode. The underlying acquisition system operated at 100 Hz, whereas the exported VO_2_, VCO_2_, and respiratory frequency values used in the analyses were breath-by-breath outputs. For the condition-by-period analyses, the respiratory measurements were summarized as arithmetic means over the 0–10, 10–20, and 20–30 min periods, as described in Section 2.3.1. The equipment was calibrated before each experiment in accordance with the manufacturer’s instructions. Because of a transient malfunction of the respiratory gas analysis system in one participant, data from the final 10 min (20–30 min) of one session were missing. Complete-case analysis was therefore applied to respiratory gas variables, yielding 29 participants. Heart rate and heart rate variability data, collected independently using the Polar H10 and unaffected by this malfunction, were analyzed for all 30 participants.

### 2.3. Data Analysis

#### 2.3.1. Data Preprocessing and Temporal Segmentation

With the exception of heart rate, all physiological variables collected during the 30 min sessions were analyzed in three periods: early (0–10 min), middle (10–20 min), and late (20–30 min). Heart rate was also summarized in 5 min intervals to depict temporal changes in greater detail. For inferential statistical testing, however, heart rate was analyzed using the same 10 min intervals as the other variables.

Heart rate variability (HRV) was analyzed from RR intervals using Kubios HRV Scientific (version 4.3.0; Kubios Oy, Kuopio, Finland). For HRV estimation, threshold-based beat correction, set to strong, was applied to all 90 recordings. The RR interval series was detrended using the smoothness priors method (λ = 500) and subsequently interpolated at 4 Hz. Power spectra were estimated using the FFT-based Welch periodogram with a 300 s window and 50% overlap [38]. Time-domain indices included the standard deviation of normal-to-normal intervals (SDNN) and the root mean square of successive differences (RMSSD). Frequency domain indices included low-frequency power (LF; 0.04–0.15 Hz) and high-frequency power (HF; 0.15–0.70 Hz). SDNN describes variability within each RR interval sample, whereas RMSSD quantifies variability between successive intervals. LF reflects a combination of physiological influences, and HF is associated with the respiratory modulation of heart rate [39]. These indices require cautious interpretation during nonstationary exercise, particularly when respiratory frequency differs across conditions.

For HRV, three non-overlapping samples were selected from each recording, corresponding to nominal periods of 0–10, 10–20, and 20–30 min from the start of the recording. SDNN, RMSSD, LF, and HF were calculated separately for each selected sample; each participant contributed one value per condition and period for each index. All 270 participant-by-condition-by-period estimates from 30 participants were retained, without additional quality-based exclusions or missing-value imputation. The nominal periods did not necessarily contain identical durations of usable RR data.

Heart rate was derived separately from the original RR intervals extracted from the KDF recording files. For each RR interval, HR (bpm) was calculated as 60,000 divided by the RR duration in milliseconds. This HR value was assigned to the time spanned by that RR interval. Consecutive, non-overlapping 1 s bins were formed from the recording start, and HR within each bin was calculated as the weighted mean of the intersecting RR-derived HR values, with weights equal to their durations of overlap with the bin.

For the condition-by-period HR analysis, each participant’s available 1 s values were averaged within the nominal 0–10, 10–20, and 20–30 min periods. Each participant contributed one HR mean per condition and period to the repeated measures analysis.

Respiratory variables included respiratory frequency, oxygen uptake (VO_2_), carbon dioxide output (VCO_2_), and derived metabolic equivalents (METs). Respiratory gas data were exported from PFT Ergo software (version 10.0; COSMED) to Microsoft Excel 365 (Microsoft Corporation, Redmond, WA, USA).For each participant and exercise condition, arithmetic means of respiratory frequency, VO_2_, VCO_2_, and METs were calculated from the exported values within the 0–10, 10–20, and 20–30 min periods. These participant-level 10 min means were used in the condition-by-period ANOVA. The separate segment-level analyses used the exported time series rather than these 10 min means (Section 2.3.2). Throughout this manuscript, metabolic responses refer to VO_2_ and VCO_2_ responses, as well as the derived MET estimates of exercise intensity. Exercise-induced carbohydrate and fat oxidation rates were not analyzed. METs were calculated by dividing mass-specific VO_2_ by the conventional resting reference value of 3.5 mL O_2_·kg^−1^·min^−1^. This reference was not based on individually measured resting values. The repeated measures ANOVAs and associated post hoc comparisons for heart rate and respiratory gas variables were performed using IBM SPSS Statistics 27 (IBM Corp., Armonk, NY, USA).

#### 2.3.2. Segment-Level Analysis of Physiological Responses by Terrain Load Direction

The content used for the moderate condition was selected for segment-level analysis because it included repeated uphill and downhill sections. Heart rate and metabolic responses were examined according to the direction of the reference terrain profile. The reference altitude profile was temporally aligned with each participant’s physiological time series using the approximate mapping described below. In the closed-loop system, cadence and software-reported power were used to determine virtual movement speed, whereas terrain gradient determined the resistance command. Although participants were instructed to maintain 50–60 rpm, this target did not ensure identical riding pace or power output across participants. The reference terrain profile was extracted from an approximately 8.3 km section of the full 12.5 km Maspalomas route and mapped to the 30 min session under the moderate condition. This section lasted approximately 18 min in the original recording, and its altitude profile served as the reference for the segment analysis. The distance and duration of the reference route do not represent the distance traveled or speed attained by individual participants.

The reference altitude profile was linearly interpolated from its ordered sample positions onto a common 1 s grid comprising 1797 samples (0–1796 s), with the first and last altitude values fixed at the grid endpoints. This grid represented the approximately 30 min exercise period. Interpolated altitude values were paired with physiological values at the corresponding elapsed times. This procedure assumed that each participant’s terrain–time trajectory could be approximated by uniformly stretching the reference profile in time. Because participant-specific route position was not recorded continuously, the actual terrain segment occupied at each physiological measurement time could not be verified. Misalignment could mix responses from adjacent climbing and descending segments, affecting the magnitude or direction of the estimated contrasts; a bias toward the null cannot be assumed. Accordingly, this analysis describes physiological changes within reference-defined segments under approximate alignment. It does not establish precise terrain–response timing, and second-by-second physiological delays were not estimated.

The resampled altitude profile was smoothed using a centered moving average extending 15 s on either side of each sample, comprising 31 samples at interior points. The rate of altitude change was calculated using central differences at interior points and one-sided differences at the endpoints, with a 1 s sample interval, and expressed in m/s. Climbing and descending segments were defined by positive and negative rates of altitude change, respectively, with an absolute rate of at least 0.025 m/s (1.5 m/min) sustained for at least 45 consecutive samples. This procedure identified 7 climbing and 8 descending segments, for a total of 15 segments in the content used for the moderate condition. The HR input for the segment-level analysis was the 1 s series derived from RR intervals as described in Section 2.3.1. For the segment-level analyses of VO_2_ and VCO_2_, each participant’s exported observations within the same rounded second were averaged, and the resulting values were linearly interpolated onto the common 1 s grid. Interpolation retained the value of the nearest endpoint outside the observed time range. A centered 15-sample moving average was applied to each participant’s heart rate (HR), VO_2_, and VCO_2_ signals. Moving-average windows were truncated at the series boundaries. The same altitude-defined segment boundaries were then applied to the smoothed physiological signals of all participants. For each participant and each classified segment, an ordinary least-squares regression slope against elapsed time in minutes was calculated for each physiological variable. Segment-level slopes were averaged separately for each participant across climbing and descending segments, with equal weight assigned to each segment within each direction. For each variable, the paired climbing and descending mean slopes were compared across participants using a two-sided Wilcoxon signed-rank test. For the VO_2_ and VCO_2_ comparisons, *p* values were calculated using SciPy (version 1.18.1) with the normal approximation without continuity correction. For HR, VO_2_, and VCO_2_, the primary effect size was the matched-pairs rank-biserial correlation, *r_rb_* = (W_+_ − W_−_)/(W_+_ + W_−_), where W_+_ and W_−_ denote the sums of the ranks of positive and negative within-participant climb-minus-descent differences; zero differences were excluded and tied absolute differences received mid-ranks [40]. This index is consistent with the Wilcoxon signed-rank test and ranges from −1 to +1. Cohen’s dz, calculated as the mean within-participant difference divided by the sample standard deviation of these differences, was additionally reported for comparability with parametric effect size conventions. Statistical significance was evaluated using the Wilcoxon test. The 95% confidence intervals for dz were obtained by inversion of the noncentral t distribution using SciPy (version 1.18.1) in Python(version 3.14). The observed t statistic was defined as dz√n, with n−1 degrees of freedom, where n is the number of participants included in the paired comparison. The resulting noncentrality limits were divided by √n to obtain the confidence limits for dz. For VO_2_ and VCO_2_, effect sizes and confidence limits were calculated from the unrounded participant-level slopes without intermediate rounding; rounding was applied only for reporting. All 30 participants were included in the heart rate analysis, whereas 29 were included in the VO_2_ and VCO_2_ analyses, as described in Section 2.2.3. Because metabolic responses may be delayed relative to load changes owing to oxygen uptake kinetics [41], the response-lag correction was varied in sensitivity analyses.

The association between the terrain gradient used to determine resistance commands and heart rate slope was also examined. The mean signed gradient (%) of each classified segment was calculated using distance-based GPX data. For each participant, segment-specific heart rate slopes were regressed on segment gradient to obtain a participant-specific coefficient, expressed as the change in heart rate slope per 1% increase in gradient (bpm/min per 1% gradient). A one-sample Wilcoxon signed-rank test was used to assess whether these coefficients differed from zero. The rank-biserial correlation for this test was calculated in the same manner using the signed ranks of the coefficients. An additional analysis was restricted to climbing segments. Examining slopes reduced reliance on absolute physiological levels, which may change over time during prolonged exercise, including through possible cardiovascular drift [30], but did not eliminate potential temporal confounding. Each participant served as the independent unit for these segment-level comparisons; individual 1 s observations were not treated as independent replicates. Sensitivity analyses varied the altitude classification threshold, the minimum segment duration, and the response-lag correction to assess their dependence on these analytical settings. The 15 s lag was evaluated as an alternative temporal alignment between the physiological series and the reference terrain profile; it did not represent a delay estimated from the RR-to-HR conversion. These analyses do not independently validate the underlying participant-specific terrain alignment.

For the high condition, altitude and heart rate data from all 30 participants were pooled to describe their static association using Pearson correlations and scatterplots. Each participant’s heart rate series was expressed as residuals after removing the participant-specific linear time trend, and altitude was expressed as residuals after removing the time trend from the common terrain profile. First differencing was also applied to reduce shared trends and serial dependence. Because detrending and differencing do not establish independence among repeated observations within participants, pooled correlations and their nominal *p* values are interpreted descriptively rather than as confirmatory participant-level inference.

#### 2.3.3. Statistical Analysis by Terrain Condition and Measurement Period

Two-way repeated-measures analysis of variance (ANOVA) was used to assess the main effects of terrain condition and measurement period, as well as their interaction. Both factors had three within-participant levels. Because all participants completed the terrain conditions in the same moderate–low–high sequence, terrain condition was confounded with visit order. Accordingly, the main condition effect represents differences across the scheduled condition visits and should not be interpreted as an isolated causal effect of terrain condition. SDNN, RMSSD, LF, and HF were natural-log-transformed from the original-scale participant-by-condition-by-period values, without intermediate rounding. HR and respiratory gas outcomes were analyzed without transformation. Descriptive HRV means and sample standard deviations are reported on the original scales, whereas HRV ANOVAs and post hoc comparisons used the natural-log scale. Normality was examined using the Shapiro–Wilk test; HRV diagnostics included log-transformed condition period values and centered orthonormal within-participant contrasts.

Mauchly’s test was used to assess sphericity. When the sphericity assumption was satisfied, results based on the original degrees of freedom were reported. When Mauchly’s test indicated a violation of sphericity (*p* < 0.05), the Greenhouse–Geisser epsilon correction was applied, and the corrected degrees of freedom and *p* values were used for interpretation.

The nominal significance level was 0.05, with multiplicity adjustments applied as specified. When a main effect or interaction was statistically significant, post hoc pairwise comparisons were interpreted using Bonferroni-adjusted *p* values. For each outcome, a comparison family comprised the three pairwise contrasts among the levels of one of the three-level factors (condition or measurement period). For simple effects, a separate comparison family was defined at each fixed level of the other factor. Adjusted *p* values were compared with 0.05; the equivalent threshold for unadjusted *p* values within a three-comparison family was 0.05/3 (approximately 0.0167). Effect sizes for the omnibus tests were reported as partial eta squared (partial η^2^). All Wilcoxon tests were two-sided. HRV pairwise comparisons used two-sided paired *t* tests on the ln scale. Main-effect comparisons averaged each participant’s ln values over the levels of the other factor.

A supplementary post hoc multiplicity analysis was performed for a family comprising the nine main-effect tests for conditions, namely, HR, SDNN, RMSSD, LF, HF, VO_2_, VCO_2_, respiratory frequency, and METs. For HRV outcomes, p values were calculated from the unrounded reanalysis statistics; for the remaining outcomes, p values were approximated from the reported F statistics and degrees of freedom in the corresponding ANOVA summary tables. Greenhouse–Geisser-corrected degrees of freedom were used where indicated. These values were compared with 0.05/9 (approximately 0.00556) by multiplying each *p* value by 9, capping the adjusted value at 1, and comparing the adjusted values with 0.05. The across-outcome correction applied only to condition main effects; period effects, condition-by-period interactions, and the exploratory segment-level Wilcoxon tests were not included in this family.

## 3. Results

Thirty healthy adult men completed the physiological evaluation of the indoor cycling system based on real-world riding footage and terrain data. All participants completed the three study visits in the fixed order of moderate, low, and high conditions. The mean interval between consecutive session start times was 53.9 ± 49.9 h. Mean cadence was 57 ± 5 rpm in the low condition, 55 ± 7 rpm in the moderate condition, and 52 ± 4 rpm in the high condition. These values represent the between-participant mean ± standard deviation of session-mean cadence values obtained from the end-of-session results screen. Although the condition-level means were within the prescribed 50–60 rpm range, these session summaries do not describe within-session cadence variability or establish individual adherence to the target. Session-mean cadence decreased as the commanded conditions became more demanding.

### 3.1. Observed Physiological Differences Across Commanded Terrain Conditions

**Figure 6:**
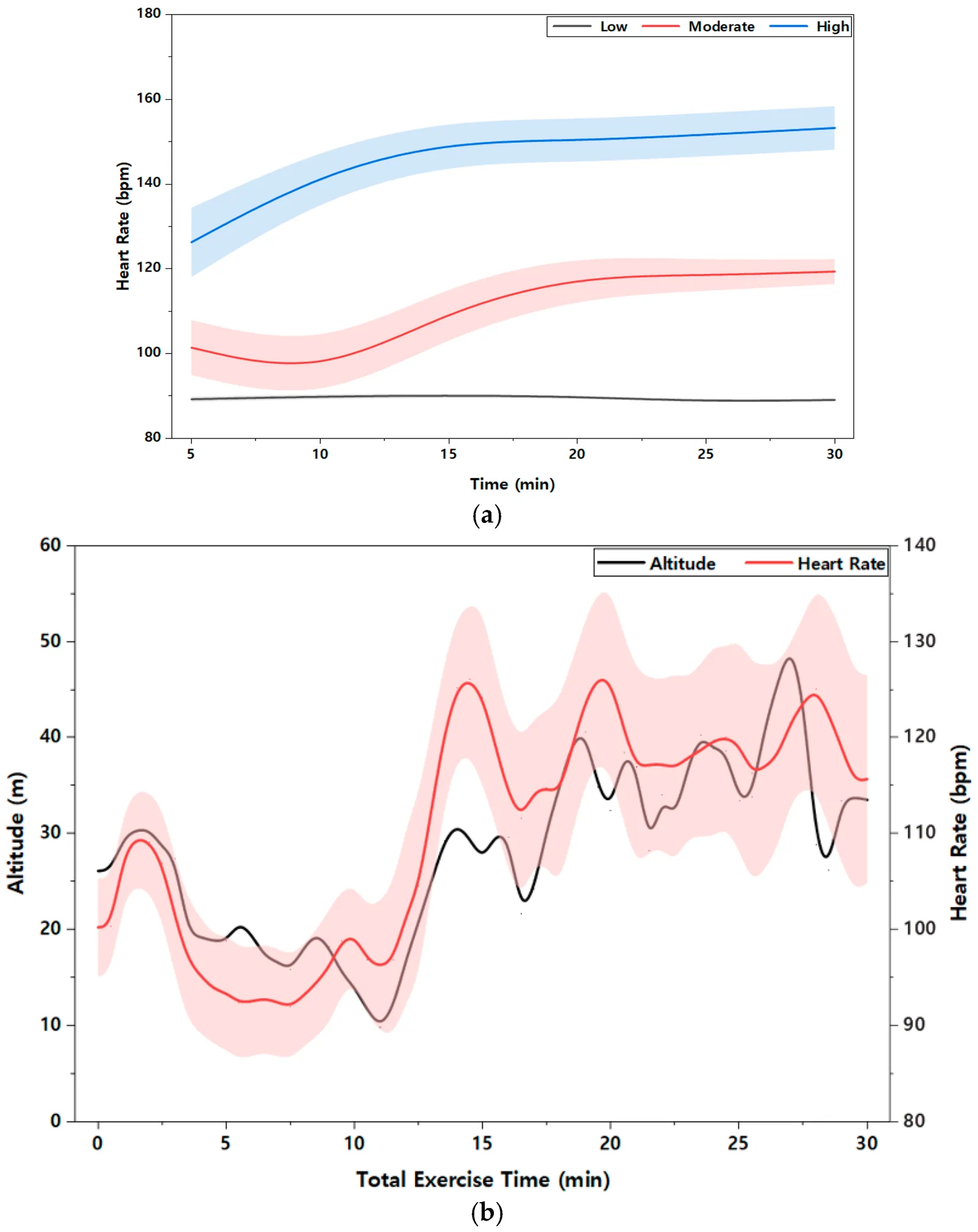

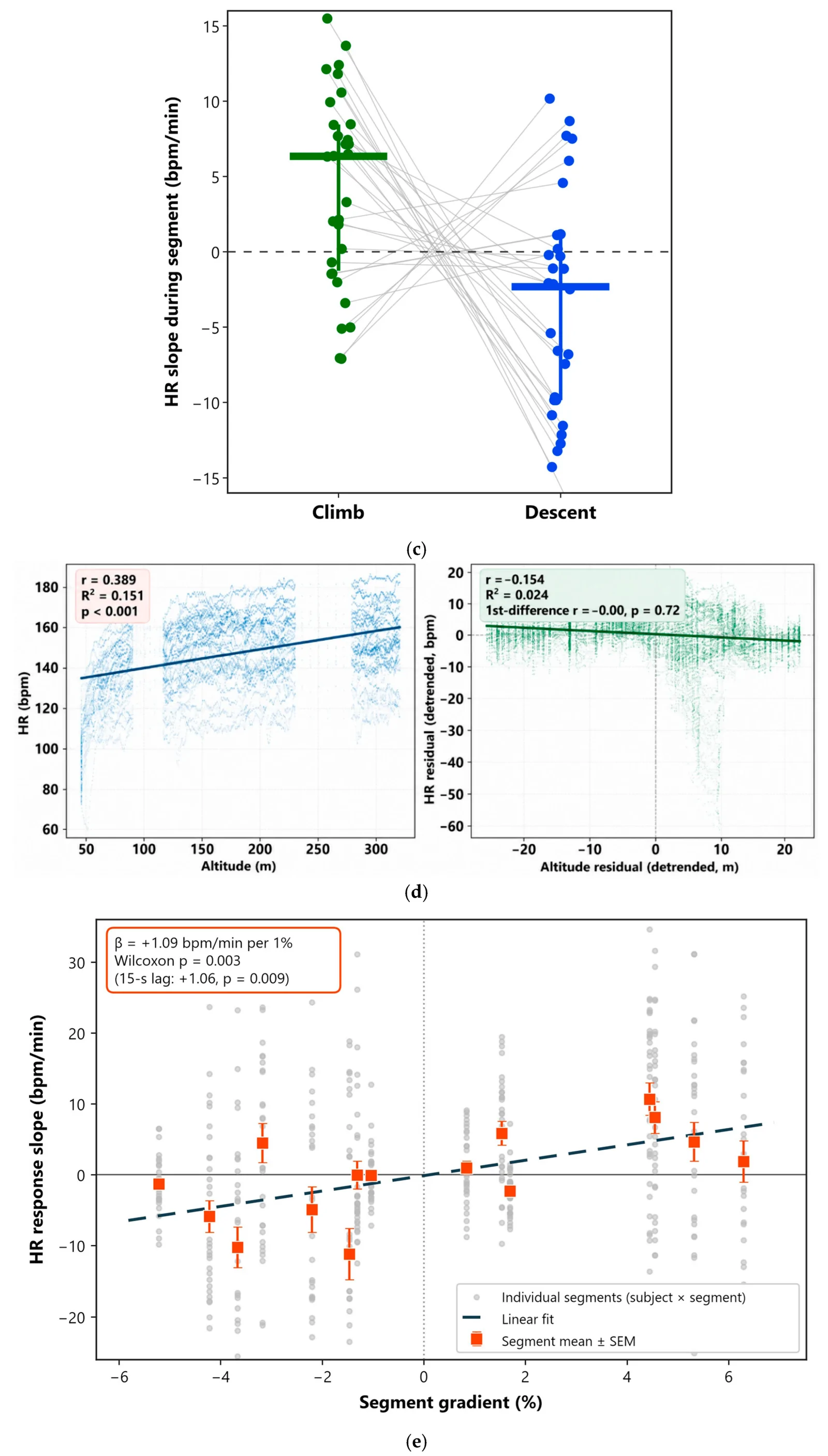
Heart rate responses across commanded terrain conditions and terrain load characteristics. (**a**) Heart rate across commanded terrain conditions. (**b**) Reference altitude profile and heart rate during the moderate condition. The reference profile was uniformly time-scaled to approximate the exercise block. Participant-specific route position was not recorded continuously, so the temporal correspondence is approximate. (**c**) Participant-level mean heart rate slopes during classified climbing and descending segments (*n* = 30). Each point represents a participant’s mean slope, with gray lines connecting the ascending and descending values for that participant. Horizontal bars indicate group medians, and vertical bars indicate interquartile ranges. Each participant’s slopes were averaged separately across seven climbing and eight descending segments. Climbing, +6.34 bpm/min (IQR, −1.26 to 8.46); descending, −2.32 bpm/min (IQR, −9.85 to 0.87); paired Wilcoxon *p* = 0.005; rank-biserial r_rb_ = 0.58; Cohen’s dz = 0.58. (**d**) Raw and detrended altitude–heart rate relationships during the high condition. The pooled correlations are descriptive, and the reported *p* values are nominal because observations within participants are not independent. (**e**) Relationship between signed segment gradient and heart rate response slope (*n* = 30). Gray circles represent individual participant segment observations; orange squares and error bars indicate segment means and standard errors of the mean (SEM), respectively. The dashed line indicates a linear fit. The mean participant-specific regression coefficient was +1.09 bpm/min per 1% gradient for the primary alignment and +1.06 bpm/min per 1% gradient with a 15 s response lag. Two-sided one-sample Wilcoxon signed-rank tests compared the participant-specific coefficients with zero (*p* = 0.003 and *p* = 0.009, respectively; rank-biserial *r_rb_* = 0.61 for the primary alignment).

Figure 6a shows heart rate trajectories analyzed in 5 min intervals across the low, moderate, and high conditions. Heart rate remained relatively stable in the low condition. In contrast, mean HR was higher in the middle and late periods than in the early period in the moderate and high conditions. Two-way repeated-measures ANOVA showed a significant main effect of terrain condition, F(2, 58) = 276.771, *p* < 0.001, partial η^2^ = 0.905; a significant main effect of measurement period, F(1.359, 39.417) = 162.859, *p* < 0.001, partial η^2^ = 0.849; and a significant condition-by-period interaction, F(2.323, 67.360) = 56.528, *p* < 0.001, partial η^2^ = 0.661. Bonferroni-adjusted post hoc tests identified significant differences among all pairs of low, moderate, and high conditions (all *p* < 0.01; Table 2 and Table 3). In the 10 min analyses, HR was higher in each successive period when averaged across conditions and within the moderate and high conditions (middle vs. late: Bonferroni-adjusted *p* = 0.0019, 0.0100, and 0.0499, respectively). In the low condition, HR was 1.12 bpm lower in the late than in the middle period (Bonferroni-adjusted *p* = 0.0202), with no significant differences in the other period comparisons. Across the physiological variables reported in Table 2 and Table 3, all main and interaction effects were significant and generally large (partial η^2^ = 0.45–0.91). In the supplementary multiplicity analysis using the updated HRV tests and the reported statistics for the remaining outcomes, all nine main effects of condition remained significant after Bonferroni adjustment within this family (all adjusted *p* values < 0.001).

**Table 2:** Significance tests for heart rate and heart rate variability parameters.

Variables	Condition	Interval			F-Value		
		0 min–10 min	10 min–20 min	20 min–30 min	Condition Effect	Period Effect	Condition × Period
		Mean ± SD	Mean ± SD	Mean ± SD			
SDNN (ms)	Low	29 ± 13	28 ± 12	29 ± 11	F(2, 58) = 246.156 *** η^2^p = 0.895	F(1.514, 43.897) = 131.481 *** (GG) η^2^p = 0.819	F(2.509, 72.753) = 43.235 *** (GG) η^2^p = 0.599
	Moderate	22 ± 10	15 ± 9	12 ± 8			
	High	8 ± 4	4 ± 2	3 ± 2			
RMSSD (ms)	Low	26 ± 16	25 ± 15	26 ± 16	F(2, 58) = 210.578 *** η^2^p = 0.879	F(1.594, 46.235) = 75.735 *** (GG) η^2^p = 0.723	F(2.592, 75.175) = 26.140 *** (GG) η^2^p = 0.474
	Moderate	20 ± 10	13 ± 8	10 ± 8			
	High	6 ± 4	3 ± 1	3 ± 1			
HR (bpm)	Low	89 ± 10	90 ± 9	89 ± 10	F(2, 58) = 276.771 *** η^2^p = 0.905	F(1.359, 39.417) = 162.859 *** (GG) η^2^p = 0.849	F(2.323, 67.360) = 56.528 *** (GG) η^2^p = 0.661
	Moderate	98 ± 10	114 ± 12	119 ± 17			
	High	134 ± 14	150 ± 16	152 ± 17			
LF (ms^2^)	Low	494 ± 579	435 ± 400	447 ± 319	F(2, 58) = 218.783 *** η^2^p = 0.883	F(2, 58) = 91.564 *** η^2^p = 0.759	F(2.959, 85.823) = 30.479 *** (GG) η^2^p = 0.512
	Moderate	309 ± 316	129 ± 180	98 ± 131			
	High	23 ± 33	9 ± 16	8 ± 16			
HF (ms^2^)	Low	501 ± 593	406 ± 445	444 ± 541	F(2, 58) = 194.903 *** η^2^p = 0.870	F(1.539, 44.625) = 97.087 *** (GG) η^2^p = 0.770	F(2.669, 77.396) = 23.760 *** (GG) η^2^p = 0.450
	Moderate	337 ± 367	156 ± 295	105 ± 173			
	High	14 ± 18	4 ± 5	3 ± 5			

***: *p* < 0.001. GG = Greenhouse–Geisser correction. Corrected degrees of freedom and *p* values are reported when Mauchly’s test indicated a violation of sphericity; otherwise, uncorrected results are shown. HR and HRV analyses included 30 participants. Values are arithmetic means ± sample SDs on the original scales (ms for SDNN and RMSSD; ms^2^ for LF and HF). All four HRV indices were natural log-transformed for inferential analysis, without intermediate rounding. HRV values represent separately calculated participant-level estimates for nominal 10 min periods. LF and HF results remain exploratory. HR means were calculated from available 1 s values (302–600 values per nominal 10 min period), without imputation.

**Table 3:** Bonferroni-adjusted post hoc tests for heart rate and heart rate variability parameters.

Variables	Post-Hoc			
	Condition Effect	Period Effect	Simple Effect: Condition Within Each Period	Simple Effect: Period Within Each Condition
ln SDNN	A > B > C	P1 > P2 > P3	All periods: A > B > C	A: n.s. B: P1 > P2 > P3 C: P1 > P2 and P3; P2 vs. P3: n.s.
ln RMSSD	A > B > C	P1 > P2 > P3	All periods: A > B > C	A: n.s. B: P1 > P2 > P3 C: P1 > P2 and P3; P2 vs. P3: n.s.
HR (bpm)	A < B < C	P1 < P2 < P3	All Periods: A < B < C	A: P2 > P3 (Δ ≈ 1 bpm); other comparisons: n.s. B, C: P1 < P2 < P3
ln LF	A > B > C	P1 > P2 > P3	All periods: A > B > C	A: n.s. B: P1 > P2 > P3 C: P1 > P2 and P3; P2 vs. P3: n.s.
ln HF	A > B > C	P1 > P2 > P3	P1: A > C and B > C; A vs. B: n.s. (adjusted *p* = 0.157) P2 and P3: A > B > C	A: n.s. B: P1 > P2 > P3 C: P1 > P2 and P3; P2 vs. P3: n.s.

A = low, B = moderate, C = high. P1 = 0–10 min, P2 = 10–20 min, P3 = 20–30 min. n.s. = not statistically significant. Note. Inequality signs indicate the actual numerical direction of statistically significant pairwise differences. Comparisons labeled n.s. do not imply numerical equality. Bonferroni correction was applied to post hoc pairwise comparisons. For HR in the low condition, only P2 versus P3 was significant; its magnitude was approximately 1 bpm. Analyses included 30 participants. HRV comparisons used natural log-transformed estimates calculated separately for each nominal 10 min period, without intermediate rounding. LF and HF comparisons are exploratory. HR comparisons used paired *t*-tests with a Bonferroni correction within each family of three pairwise comparisons. HR P2–P3 adjusted *p* values were 0.0202 (low), 0.0100 (moderate), and 0.0499 (high).

### 3.2. Segment-Level Heart Rate Responses by Reference Terrain Classification

The content used for the moderate condition, which included repeated uphill and downhill sections (Figure 4b), was used to examine heart rate changes during the session. Sustained climbing and descending segments were classified from the slope of the smoothed reference altitude trajectory. Heart rate slopes (bpm/min) were estimated within each non-overlapping segment and averaged separately within each participant for climbing and descending segments. These paired participant-level means were then compared. This analysis assessed differences between reference-defined segment types under the approximate temporal mapping described in Section 2.3.2; it did not estimate precise physiological delays or directly measure delivered mechanical workload.

Figure 6b displays the reference altitude profile and heart rate on a common time axis during the session under the moderate condition. Heart rate generally increased during intervals corresponding to sustained altitude increases in the reference profile and decreased or increased more slowly during some descending intervals or transitions. This visual pattern describes an apparent association under the adopted alignment and does not verify the terrain segment occupied by each participant at a given time.

In the segment-level analysis (Figure 6c), the median heart rate slope during classified climbing segments was +6.34 bpm/min (interquartile range [IQR], −1.26 to 8.46), compared with −2.32 bpm/min (IQR, −9.85 to 0.87) during classified descending segments. The paired contrast was statistically significant (two-sided Wilcoxon signed-rank test, *p* = 0.005; rank-biserial *r_rb_* = 0.58; Cohen’s dz = 0.58, 95% CI 0.19–0.96). Of the 30 participants, 21 (70%) had higher mean HR slopes during classified climbing segments than during classified descending segments; the remaining nine participants (30%) did not show this ordering. This comparison does not require that the climbing slope be positive and the descending slope be negative in the same participant. Participant-level climbing and descending slopes were negatively correlated (r ≈ −0.93). This correlation describes related summaries from the same session and is not independent evidence that cardiovascular drift or temporal misalignment was excluded.

The absence of continuous participant-specific route-position records introduces uncertainty in assigning physiological observations to terrain segments. Depending on the temporal structure of the signals, misalignment could affect both the magnitude and direction of the estimated climb–descent contrast. In the reported sensitivity analyses, perturbing temporal alignment preserved the direction of the group contrast while reducing its magnitude. This result reflects sensitivity to the examined perturbations; it does not validate the original alignment. Under the system’s intended control scheme, climbing increased the resistance command, whereas descending returned it toward its minimum.

Segment-specific heart rate slopes were also regressed on mean signed segment gradient (%) (Figure 6e). The mean participant-specific regression coefficient was +1.09 bpm/min per 1% gradient and differed from zero (two-sided one-sample Wilcoxon signed-rank test, *p* = 0.003; rank-biserial *r_rb_* = 0.61). The association appeared to be driven mainly by the contrast between climbing and descending segments; the association restricted to climbing segments did not reach statistical significance (*p* = 0.067). Its direction was retained under the altitude classification thresholds, minimum segment durations, and response-lag corrections examined. These results describe an association with signed gradient under the adopted alignment and do not establish a calibrated mechanical dose–response relationship or precise physiological latency.

When altitude and heart rate data from all 30 participants were pooled for the high condition (Figure 6d), the raw correlation was r = 0.389 (R^2^ = 0.151). After the removal of the shared linear time trend, the correlation was r = −0.154 (R^2^ = 0.024). The first-differenced analysis yielded a coefficient near zero (r = 0.00; nominal *p* = 0.72). Because the pooled observations retained within-participant dependence, these results are presented descriptively and are not treated as confirmatory evidence of terrain tracking.

### 3.3. Segment-Level Metabolic Responses by Reference Terrain Classification

VO_2_ and VCO_2_ responses were examined using the same reference-defined segments in the moderate condition. The median VO_2_ slope was +126 mL·min^−2^ during classified climbing segments (IQR, −20 to 275) and −102 mL·min^−2^ during classified descending segments (IQR, −333 to 40). The paired contrast was statistically significant (two-sided Wilcoxon signed-rank test, *p* = 0.016; rank-biserial *r_rb_* = 0.51; Cohen’s dz = 0.48, 95% CI 0.09–0.86), and the participant-level mean climbing slope exceeded the corresponding mean descending slope in 20 of 29 participants (69%).

VCO_2_ showed a contrast in the same direction. The median VCO_2_ slope was +132 mL·min^−2^ during classified climbing segments (IQR, −33 to 190) and −121 mL·min^−2^ during classified descending segments (IQR, −191 to 59). The paired contrast was statistically significant (two-sided Wilcoxon signed-rank test, *p* = 0.004; rank-biserial *r_rb_* = 0.61; Cohen’s dz = 0.59, 95% CI 0.19–0.98), and the participant-level mean climbing slope exceeded the corresponding mean descending slope in 19 of 29 participants (66%). These percentages describe the ordering of each participant’s mean slopes. They do not establish physiological responder status or require positive climbing and negative descending slopes in the same participant. Responses varied substantially between participants. Climbing and descending slopes were negatively correlated (VO_2_, r ≈ −0.93; VCO_2_, r ≈ −0.95); these correlations describe related within-session summaries and do not independently validate terrain alignment or identify the underlying response mechanism.

With a minimum segment duration of 45 s, HR, VO_2_, and VCO_2_ contrasts remained significant across the classification thresholds examined. When the minimum duration was shortened to 30 s, the VCO_2_ contrast remained significant, although the strength of evidence decreased. These findings depend on the duration of the classified segments and should be interpreted within the specific settings examined. Delayed metabolic adjustment may contribute to differences between shorter and longer segments [41], but the present analysis did not directly estimate the magnitude or timing of physiological delays.

**Figure 7:**
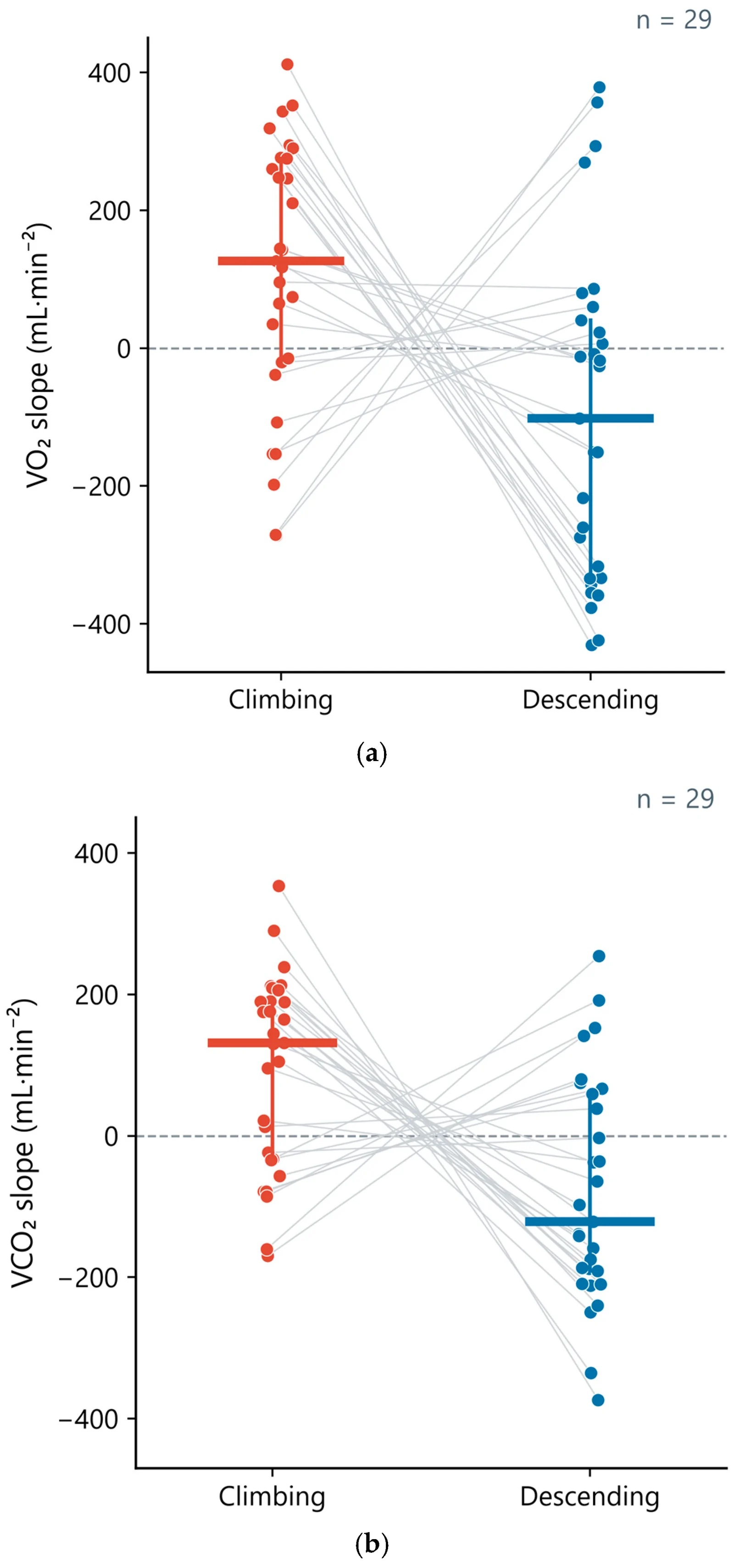

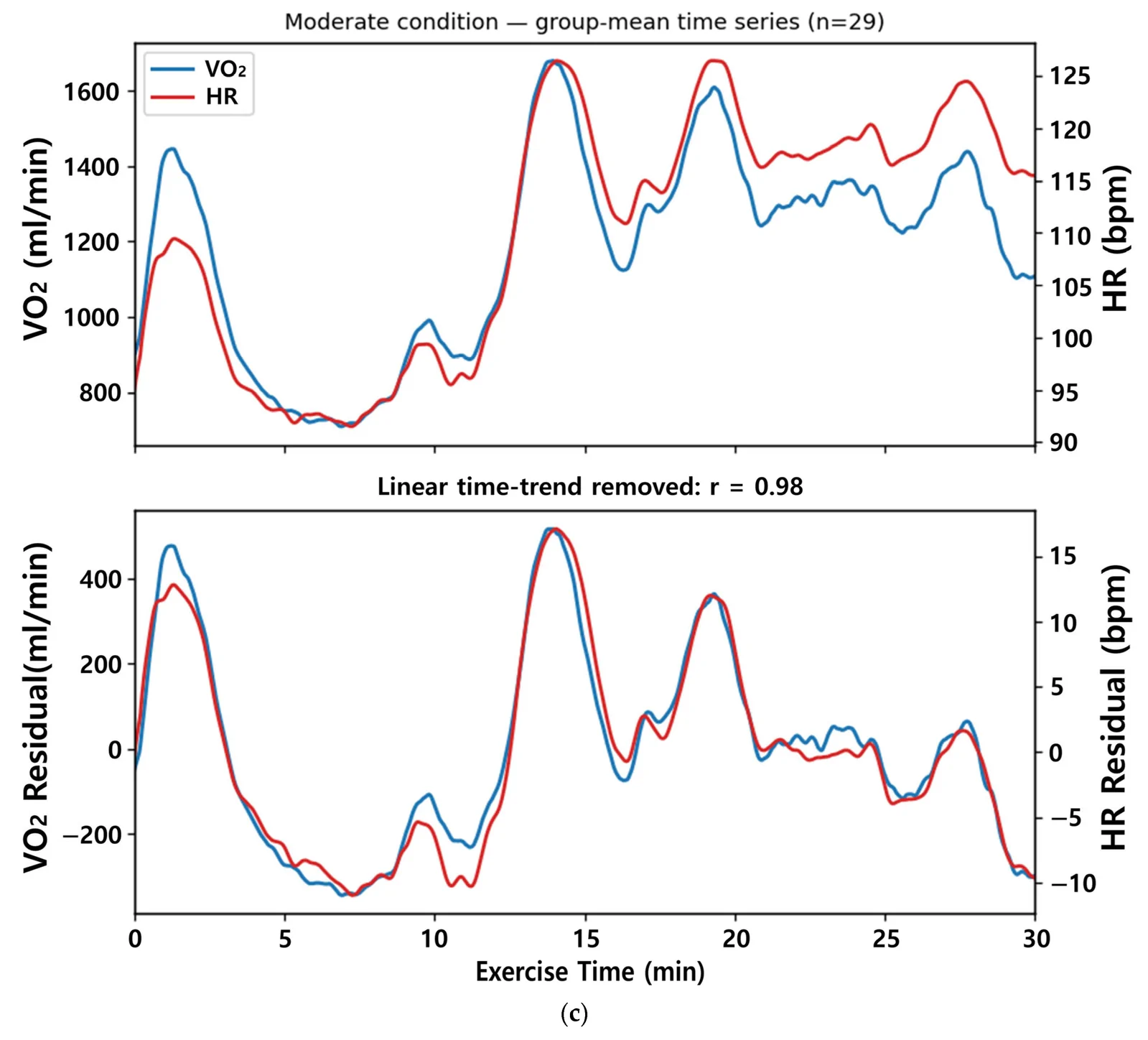
Segment-level respiratory gas analyses during the moderate condition. Panels (**a**,**b**) show participant-level slopes for climbing and descending segments. Panel (**c**) shows group mean VO2 and heart rate time series before and after removal of the common linear time trend (detrended r = 0.98). The correlation in panel (**c**) describes temporal similarity between the processed signals and is interpreted descriptively. (**a**) Participant-level VO_2_ slopes during climbing and descending segments. Each point represents one participant’s mean slope across seven climbing or eight descending segments (*n* = 29), and gray lines connect paired values. Horizontal bars and whiskers indicate the median and IQR. Climbing, +126 mL·min^−2^; descending, −102 mL·min^−2^; Wilcoxon p = 0.016; rank-biserial *r_rb_* = 0.51; Cohen’s dz = 0.48. (**b**) Participant-level VCO_2_ slopes during climbing and descending segments. Each point represents one participant’s mean slope across seven climbing or eight descending segments (*n* = 29), and gray lines connect paired values. Horizontal bars and whiskers indicate the median and IQR. Climbing, +132 mL·min^−2^; descending, −121 mL·min^−2^; Wilcoxon p = 0.004; rank-biserial *r_rb_* = 0.61; Cohen’s dz = 0.59. (**c**) Temporal association between oxygen uptake and heart rate.

Descriptive VO_2_–HR correlations were calculated for the moderate condition after linear detrending of the processed time series (Figure 7c). The group mean correlation was r = 0.98, and the median participant-specific correlation was r = 0.94 (IQR, 0.93–0.95). These coefficients describe temporal similarity between the processed signals. Because smoothing and residual serial dependence can influence these estimates, they are not interpreted as independent confirmation of physiological coupling or terrain tracking.

### 3.4. Heart Rate Variability

Time domain indices (SDNN and RMSSD) and frequency-domain indices (LF and HF) were calculated to characterize HRV responses across the commanded terrain conditions.

All four HRV indices were analyzed after natural-log transformation. Significant main effects of terrain condition and measurement period, and significant condition-by-period interactions, were observed for SDNN, RMSSD, LF, and HF (all *p* < 0.001; Table 2). Averaged across measurement periods, each index was higher in the low than in the moderate condition, and higher in the moderate than in the high condition. Averaged across conditions, all indices decreased from P1 to P2 and from P2 to P3 (Bonferroni-adjusted *p* < 0.05; Table 3).

Within each measurement period, SDNN, RMSSD, and LF differed among all three conditions, with low > moderate > high. For HF, the low and moderate conditions both exceeded the high condition in P1, but the low–moderate comparison was not significant (Bonferroni-adjusted *p* = 0.157). All three conditions differed in P2 and P3, with low > moderate > high (Table 3).

Within the low condition, no pairwise period differences were significant for any HRV index. Within the moderate condition, all four indices decreased from P1 to P2 and from P2 to P3. Within the high condition, P1 exceeded P2 and P3, whereas P2 and P3 did not differ significantly (Bonferroni-adjusted comparisons; Table 3). Nonsignificant comparisons do not establish equivalence or the absence of change. The LF and HF findings remain exploratory and do not support direct quantitative inferences about autonomic regulation.

### 3.5. Respiratory Gas Exchange

Respiratory frequency, VO_2_, VCO_2_, and METs were analyzed to evaluate respiratory and metabolic responses across the commanded terrain conditions.

Respiratory frequency remained relatively stable across measurement periods in the low condition and increased across the moderate and high conditions (Table 4). In the high condition, respiratory frequency was highest from the first measurement period and showed the largest increase over time. VO_2_ and VCO_2_ were low and relatively stable in the low condition, increased from the early to the middle period in the moderate condition, and were high across all three measurement periods in the high condition. Across the three measurement periods, METs were approximately 2.5 in the low condition, 3.7–5.1 in the moderate condition, and 6.9–7.7 in the high condition (Table 4).

All respiratory gas variables satisfied the normality assumption and were analyzed without transformation. Significant main effects of terrain condition and measurement period, and significant interactions between the two factors, were observed for respiratory frequency, VO_2_, VCO_2_, and METs (all *p* < 0.01; Table 4 and Table 5).

**Table 4:** Significance tests for respiratory gas and metabolic parameters.

Variables	Condition	Interval			F-Value		
		0 min–10 min	10 min–20 min	20 min–30 min	Condition Effect	Period Effect	Condition × Period
VO_2_ (mL/min)	Low	651 ± 76	645 ± 45	651 ± 48	F(1.265, 35.420) = 455.345 ** (GG) η^2^p = 0.942	F(2, 56) = 129.971 ** η^2^p = 0.823	F(2.889, 80.880) = 45.759 ** (GG) η^2^p = 0.620
	Moderate	964 ± 68	1315 ± 72	1301 ± 69			
	High	1801 ± 110	1991 ± 136	1976 ± 147			
VCO_2_ (mL/min)	Low	578 ± 70	571 ± 43	569 ± 45	F(1.255, 35.130) = 439.814 ** (GG) η^2^p = 0.940	F(2, 56) = 90.041 ** η^2^p = 0.763	F(2.676, 74.922) = 38.967 ** (GG) η^2^p = 0.582
	Moderate	892 ± 78	1307 ± 83	1294 ± 82			
	High	1791 ± 124	1969 ± 143	1915 ± 154			
Rf (breaths/min)	Low	18 ± 2	18 ± 2	18 ± 2	F(1.380, 38.630) = 74.023 ** (GG) η^2^p = 0.726	F(1.351, 37.824) = 57.208 ** (GG) η^2^p = 0.671	F(2.399, 67.180) = 15.213 ** (GG) η^2^p = 0.352
	Moderate	21 ± 2	22 ± 2	24 ± 3			
	High	26 ± 3	30 ± 4	32 ± 4			
METs	Low	2.5 ± 0.3	2.5 ± 0.2	2.5 ± 0.2	F(1.130, 31.646) = 357.047 ** (GG) η^2^p = 0.927	F(2, 56) = 89.734 ** η^2^p = 0.762	F(2.316, 64.862) = 43.586 ** (GG) η^2^p = 0.609
	Moderate	3.7 ± 0.3	5.1 ± 0.4	5.0 ± 0.5			
	High	6.9 ± 0.6	7.7 ± 0.7	7.6 ± 0.7			

**: *p* < 0.01. GG = Greenhouse–Geisser Correction. When sphericity was violated, Greenhouse–Geisser-corrected degrees of freedom and *p* values were reported; otherwise, results based on sphericity-assumed degrees of freedom were used. Respiratory gas analyses included 29 participants.

**Table 5:** Bonferroni-adjusted post hoc tests for respiratory gas and metabolic parameters.

Variables	Post-Hoc			
	Condition Effect	Period Effect	Simple Effect: Condition Within Each Period	Simple Effect: Period Within Each Condition
VO_2_ (mL/min)	A < B < C	P1 < P2 and P3; P2 vs. P3: n.s.	All Periods: A < B < C	A: n.s. B, C: P1 < P2 and P3; P2 vs. P3: n.s.
VCO_2_ (mL/min)	A < B < C	P1 < P2 and P3; P2 vs. P3: n.s.	All Periods: A < B < C	A: n.s. B, C: P1 < P2 and P3; P2 vs. P3: n.s.
Rf (breaths/min)	A < B < C	P1 < P2 < P3	All Periods: A < B < C	A: n.s. B: P1 < P2 < P3 C: P1 < P2 < P3
METs	A < B < C	P1 < P2 and P3; P2 vs. P3: n.s.	All Periods: A < B < C	A: n.s. B, C: P1 < P2 and P3; P2 vs. P3: n.s.

A = low, B = moderate, C = high. P1 = 0–10 min, P2 = 10–20 min, P3 = 20–30 min. n.s. = not statistically significant. Note. Inequality signs indicate the actual numerical direction of statistically significant pairwise differences. Comparisons labeled n.s. do not imply numerical equality. Bonferroni correction was applied to post hoc pairwise comparisons. Respiratory gas analyses included 29 participants.

## 4. Discussion

This study characterized cardiovascular, heart rate variability, and metabolic responses to three commanded terrain load conditions in a real-world, video-based indoor cycling system. Heart rate, respiratory frequency, VO_2_, VCO_2_, and METs increased across the low, moderate, and high conditions, whereas HRV indices decreased. In the moderate condition, participant-level physiological slopes differed between segments classified as climbing and descending. The condition-level findings describe physiological differences across the tested conditions under the fixed-order protocol; the interpretation of the segment-level associations also depends on the approximate alignment between terrain and physiological data.

### 4.1. Interpretation of Physiological Responses Across Commanded Terrain Conditions

Metabolic demand was approximately 2.5 METs in the low condition, 3.7–5.1 METs in the moderate condition, and 6.9–7.7 METs in the high condition. These values span light, moderate, and vigorous absolute-intensity categories [3,11]. Physiological responses differed across the three commanded conditions under the fixed-order protocol in the participants studied. These group-level observations do not establish individualized intensity targets or the reliability of responses across other routes or repeated exposures. No statistically significant differences in respiratory and metabolic variables were detected across the three 10 min periods in the low condition; this does not establish equivalence across periods. This result does not characterize moment-to-moment fluctuations in delivered mechanical load. The fixed 1-MET reference does not account for interindividual variation in resting oxygen uptake [42]. Thus, these values describe exercise intensity relative to a conventional reference and should not be interpreted as measured multiples of each participant’s resting metabolism. This limits individual-level interpretation; the magnitude and direction of the discrepancy for the present sample are unknown.

### 4.2. Segment-Level Associations Between Terrain Classification and Heart Rate Response

The segment-level analysis compared each participant’s mean heart rate slope across intervals classified as climbing and descending. The climbing slope exceeded the descending slope in 70% of participants. The paired slope contrast had a rank-biserial correlation (*r_rb_*) of 0.58 (Cohen’s dz = 0.58). The percentage refers to the ordering of the slopes and does not require both a positive climbing slope and a negative descending slope in each participant. The direction of the group contrast was retained under the sensitivity analysis settings examined. However, substantial individual variation and approximate alignment with terrain limit the interpretation of participant-level responses.

Across the classified segments, the estimated heart-rate slope increased by approximately 1.1 bpm/min per 1% increase in signed gradient (Figure 6e). This association appeared to be driven mainly by the contrast between climbing and descending segments; the association restricted to climbing segments did not reach statistical significance. The result, therefore, describes an association with signed terrain gradient under the adopted alignment procedure. It does not establish a calibrated relationship between delivered mechanical workload and physiological response.

The actuator was designed to increase resistance during climbing and return to its minimum during descending. Descending segments can consequently be interpreted as intended periods of load relief and recovery, rather than as a reproduction of all mechanical conditions encountered during outdoor descent. Previous comparisons of uphill and flat cycling at similar intensity provide context for terrain-related physiological responses [43]. In the present study, however, the analytical input was commanded gradient, and the delivered mechanical power was not validated. The segment-level findings, therefore, concern associations with commanded terrain conditions; the magnitude of the actual change in mechanical work remains unquantified.

### 4.3. Time Trends in the Altitude–Heart Rate Association Under the High Condition

In the high condition, altitude increased almost linearly with exercise time (altitude–time r ≈ 0.99), while HR also increased during the session. An upward trend in heart rate alone is insufficient to confirm cardiovascular drift. Experimental studies of this phenomenon have assessed HR together with stroke volume during prolonged exercise [44]. The present analysis did not establish a constant delivered mechanical workload or include concurrent stroke volume measurements to identify that mechanism. Changes in exercise demand, response kinetics, and other time-dependent influences remain possible contributors. Shared trends can generate correlations between nonstationary series [31,32]. The raw altitude–HR correlation (r = 0.389) decreased in magnitude after linear detrending (r = −0.154), and the first-differenced coefficient was near zero (nominal *p* = 0.72). These descriptive findings are consistent with a contribution from the shared time trend; they do not demonstrate the occurrence or removal of physiological cardiovascular drift. First differencing also does not resolve all dependence among pooled repeated observations. The moderate condition slope comparisons likewise do not establish that drift was absent or eliminated.

### 4.4. Cardiovascular and Metabolic Patterns Within the Moderate Condition

The condition-level comparisons describe overall physiological differences, while the segment-level analyses describe variation within the moderate session. The latter provides information beyond 10 min averages, but they remain observational associations within a single terrain condition. Shared segment classification also means that HR, VO_2_, and VCO_2_ analyses are subject to the same uncertainty in terrain alignment.

The 15 s smoothed VO_2_ series showed repeated increases and decreases within the session, despite the broader increase from the early to the middle measurement period. Participant-level VO_2_ and VCO_2_ slopes were greater during intervals classified as climbing than during intervals classified as descending at the group level. These findings are consistent with physiological responses to changes in exercise demand. Their attribution to specific terrain events is limited by the absence of participant-specific route-position records and validated mechanical workload measurements.

After linear detrending, VO_2_ and heart rate remained highly correlated in the group mean series (r = 0.98) and at the participant level (median r = 0.94). These coefficients describe temporal similarity in the processed signals. Smoothing and residual serial dependence can influence their magnitude, and the removal of a linear trend does not establish independence among successive observations. The correlations should therefore be interpreted descriptively, without treating them as an independent confirmation of physiological coupling or terrain tracking. Similarly, the inverse correlations between climbing and descending slopes describe related summaries from the same session and do not independently resolve uncertainty about alignment or response mechanisms.

Visual information and perceived effort may influence cardiovascular responses during cycling [19,20,25,26]. The accompanying VO_2_ and VCO_2_ patterns broaden the physiological characterization, but this design cannot separate mechanical, visual–perceptual, and behavioral contributions. Cadence was prescribed at 50–60 rpm, and the available group means do not establish continuous adherence by each participant. Cadence variation within the terrain-defined segments could have contributed to the HR and VO_2_ slopes; this possibility could not be evaluated or adjusted for because continuous cadence recordings were unavailable. Comparator conditions and synchronized measurements of cadence, mechanical workload, and route position are needed to distinguish these contributions.

### 4.5. Heart Rate Variability During Exercise

SDNN and RMSSD were highest in the low condition and decreased across the moderate and high conditions. These findings are consistent with exercise-related reductions in heart rate variability (HRV) reported in previous studies [10,45,46]. LF and HF power also decreased as the commanded condition became more demanding. LF is not a specific measure of cardiac sympathetic activity; therefore, its reduction cannot be attributed to a single branch of the autonomic nervous system [47]. The interpretation of frequency domain results obtained during dynamic exercise also requires the consideration of nonstationarity and respiratory changes. Accordingly, the observed spectral differences reflect variation in the analyzed HRV indices and should not be interpreted as direct measurements of sympathetic or parasympathetic activity.

Mean respiratory frequencies were approximately 26–32 breaths/min (0.43–0.53 Hz) in the high condition and 21–24 breaths/min (0.35–0.40 Hz) in the moderate condition. These condition-level means fall within the extended 0.15–0.70 Hz HF band used in the reanalysis. Their inclusion within the band does not establish that respiratory-related variability was fully captured for every participant, nor does it eliminate the influence of respiration and exercise nonstationarity on HF estimates. The extended band definition should also be considered when comparing these values with results obtained using the conventional 0.15–0.40 Hz band [39]. Accordingly, HF power is not interpreted as a direct quantitative measure of cardiac parasympathetic activity. Low absolute HRV values alone also do not establish that the recording system reached its lower detection limit.

### 4.6. Limitations and Future Directions

All participants completed the conditions in the same moderate–low–high sequence, precluding the separation of condition effects from visit-order or carryover effects. The observed between-condition differences may therefore reflect adaptation to the equipment, learning, or the influence of preceding sessions; however, the direction and magnitude of these contributions cannot be determined from the present study. Because the high condition was always assessed last, residual effects from preceding sessions may have contributed to its estimated effect. The shortest interval between visits was 17.4 h, leaving less than 24 h between the start times of consecutive sessions. Incomplete recovery from a preceding session may consequently have contributed to between-condition differences in HRV and other variables, although its contribution cannot be quantified from the present analyses. Session start times also varied among participants, and no separate familiarization session was conducted; thus, time-of-day and learning effects cannot be ruled out. As no meal restrictions were imposed, differences in pre-exercise nutritional state also remain a potential source of response variability. The climbing–descending comparison within the moderate condition was conducted within the same session and was therefore less directly affected by visit order than the between-condition comparisons. Nevertheless, elapsed time within the session, preceding segments, cadence variation, and uncertainty in terrain alignment remain relevant to this comparison.

The study did not include conditions with asynchronous visual information and mechanical loading, fixed-resistance exercise, or conventional indoor cycling. It therefore cannot isolate the effect of visual–mechanical synchronization or establish a clinical advantage over other exercise modalities. The findings characterize responses to the three commanded conditions tested. Evaluating the contribution of synchronization would require a within-participant comparison incorporating appropriate control conditions. No direct comparison with Zwift or another commercial platform was performed. Consequently, differences in physiological responses, immersion, and resistance-control accuracy between platforms remain untested.

The principal measurement limitations concern mechanical workload and temporal alignment. Software-reported power was not independently validated as a measure of delivered mechanical workload; commanded gradient was therefore used as the load-related input. Accordingly, the analysis does not validate the actual mechanical work rate. In addition, the approximately 18 min reference terrain profile was uniformly stretched in time to approximate the 30 min exercise block and applied to participants without continuous route-position logging. Detailed participant-specific virtual speed records were unavailable, preventing the comparison of the nominal mean speed implied by this mapping with individual progression and the quantification of speed variation within and between conditions. Differences in individual progression may have affected both segment classification and the estimated physiological contrasts. The direction and magnitude of this potential bias cannot be determined from the available alignment information. Although the direction of the effect was retained under the reported sensitivity analysis settings, these analyses do not establish that the reference profile matched each participant’s actual trajectory. Validated workload measurements and continuous route-position logging are needed to more precisely assess terrain-related responses.

Of the 30 participants, 9 (30%) did not exhibit a higher mean HR slope in classified climbing segments than in classified descending segments. This finding does not mean that HR failed to rise during every climb or that these participants lacked a cardiovascular response. Research using cycle ergometers has demonstrated substantial interindividual variation in the gain and time course of HR responses to changes in workload [48]. Delayed adjustment and incomplete recovery from preceding segments may therefore affect the slope estimated for a subsequent segment. Fitness-related differences in adjustment and recovery offer another possible explanation, but these mechanisms were not identified in the present analysis. Their contributions cannot be distinguished from those of uncertainty in terrain alignment and variation in cadence. The absence of continuous cadence recordings precluded the calculation of time spent within the prescribed 50–60 rpm range and the assessment of cadence during the segments classified from the reference terrain profile. The contribution of cadence variation to the slope contrasts, therefore, remains uncertain, particularly given the lower session-mean cadence in the more demanding conditions. Accordingly, this finding is interpreted as heterogeneity in the estimated slope contrast rather than evidence of a distinct physiological nonresponder subgroup.

Additional limitations concern signal processing and statistical dependence. The detrended VO_2_–HR correlations remain descriptive because linear detrending did not fully address the effects of smoothing and serial dependence. The pooled analysis of the high condition also retained a repeated-observation structure after first differencing. Stronger inference requires methods that account for within-participant dependence and serial correlation.

The recruitment target was informed by participant numbers in previous studies rather than an effect-size assumption for a prespecified primary contrast. Those studies differed in their populations, interventions, and outcomes, so their sample sizes do not establish statistical adequacy for the present design. The separate sensitivity analysis was conditional on an assumed repeated-measures correlation of 0.50, ε = 1, and a nominal α level of 0.05. It did not incorporate adjustment across multiple outcomes and does not establish 80% power for the condition-by-period interaction, individual pairwise comparisons, or the exploratory climbing–descending contrasts. Respiratory gas analyses included 29 participants, whereas the sensitivity analysis assumed 30. The precision of the segment-level estimates and estimates of response heterogeneity may therefore be limited. Future studies should prespecify the primary contrast and justify sample size using appropriate effect-size and within-participant variance assumptions, together with the planned multiplicity adjustment.

The sample consisted of 30 healthy young adult men aged 18–27 years, limiting generalization across age, sex, fitness, and health status. Responses and safety in older adults, women, and people with chronic disease were not evaluated. The activity descriptors were self-reported after measurement and did not quantify aerobic fitness or cycling training status. Only one video was tested per condition; therefore, generalizability to other videos representing the same terrain category or to the full 62-video library remains untested. The study also did not assess RPE, cybersickness, immersion, fatigue, or usability. Its short-term laboratory design provides no evidence about sustained adherence, training adaptations, or clinical outcomes. These questions require studies that include more diverse participants, measures of subjective experience, and longitudinal follow-up.

The upright indoor cycle configuration and prescribed cadence of 50–60 rpm also limit generalization to road cycling, particularly competitive cycling. The protocol did not reproduce the range of postures and self-selected cadences used outdoors; posture can alter muscle recruitment [8]. Each exercise session lasted 30 min, and the three 10 min periods were analysis windows within that session. Fatigue and discomfort were not assessed. Consequently, the findings do not establish the physiological effects of cumulative fatigue or prolonged exposure to this posture and cadence during longer road cycling sessions.

## 5. Conclusions

Cardiovascular, HRV, and metabolic responses differed across the three commanded terrain conditions under the fixed-order protocol in healthy young men. In the moderate condition, participant-level HR, VO_2_, and VCO_2_ slopes differed between climbing and descending intervals, with substantial individual variation. These findings characterize physiological responses under the tested protocol and identify segment-level associations for further investigation.

The delivered mechanical workload was not validated, and terrain response alignment relied on a uniformly time-scaled reference profile without continuous participant-specific route position logging. Because all participants completed the conditions in the same fixed order, condition effects cannot be separated from visit-order effects. The findings, therefore, do not establish mechanical load accuracy, precise terrain tracking, or an independent benefit of visual–mechanical synchronization. Future studies should combine validated workload and route position measurements with appropriate comparator conditions and analyses that account for serial dependence. Frequency domain HRV findings remain exploratory because exercise nonstationarity, respiratory variability, and unassessed sensitivity to recording quality limit their physiological interpretation.

## Figures and Tables

**Table 1:** Specifications of the real-world video-based indoor cycling system. * Power denotes the software-reported output; it was not validated as the delivered mechanical workload in this experiment.

**Specification**	**Details**	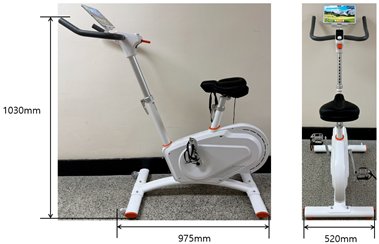
**Dimensions**	975 (L) mm × 520 (W) mm × 1030 (H) mm	
**Mass**	30 kg	
**Maximum Torque**	32 N·m	
**Maximum Power**	402 W	
**Maximum Cadence**	120 RPM	
**Reported Data**	Cadence; software-reported power *	
**Flywheel Mass**	7 kg	
**Resistance System**	Electromagnetic Resistance	
**Operating Mode**	Power mode, Gradient mode, Resistance mode	
**User Height Range**	155~185 cm	
**Maximum User Mass**	115 kg	
**Wireless Communication**	Bluetooth	

## Data Availability

The data presented in this study are only available upon request from the corresponding author. The data are not publicly available as they contain information that could compromise the privacy of the study’s participants.
